# Understanding visual attention in childhood: Insights from a new visual foraging task

**DOI:** 10.1186/s41235-016-0016-5

**Published:** 2016-11-14

**Authors:** Inga María Ólafsdóttir, Tómas Kristjánsson, Steinunn Gestsdóttir, Ómar I. Jóhannesson, Árni Kristjánsson

**Affiliations:** Department of Psychology, School of Health Sciences, University of Iceland, 101 Reykjavík, Iceland

**Keywords:** Executive Function, Visual Search, Visual Attention, Switch Cost, Work Memory Capacity

## Abstract

A recently developed visual foraging task, involving multiple targets of different types, can provide a rich and dynamic picture of visual attention performance. We measured the foraging performance of 66 children aged 4–7 years, along with measures of two conceptually related constructs, self-regulation and verbal working memory. Our results show that foraging patterns of young children differ from adult patterns. Children have difficulty with foraging for two target types, not only when they are defined by a conjunction of features but, unlike adults, also when they forage simultaneously for two target types that are distinguished from distractors by a single feature. Importantly, such feature/conjunction differences between adults and children are not seen in more traditional single-target visual search tasks. Interestingly, the foraging patterns of the youngest children were slightly more adult-like than of the oldest ones, which may suggest that older children attempt to use strategies that they have not yet fully mastered. The older children were, however, able to complete more trials, during both feature and conjunction foraging. Self-regulation and verbal working memory did not seem to affect foraging strategies, but both were connected with faster and more efficient foraging. We propose that our visual foraging paradigm is a promising avenue for studying the development of visual cognitive abilities.

## Significance

A new foraging task reveals that children have trouble foraging for targets defined by two different features; a task with which adults have little trouble with. Traditional single-target visual search tasks do not reveal these development-based differences in attentional abilities. The foraging task shows interesting overlap with measures of cognitive control abilities in children and may through further development work as a proxy for more general functions than visual processing and attention. Ease of testing and straightforward data interpretation make the task an attractive option for tests of visual attention.

## Background

Single-target visual search (Wolfe, 1998) has long been among the main methods for assessing visual attention. In single-target search tasks, participants must find a predetermined target among distractor sets of various sizes (see e.g. Bravo & Nakayama, 1992; Treisman & Gelade, 1980). When the target is defined by a single feature, such as a green disc among red discs, participants tend to respond quickly regardless of set-size. But when the target is defined by a conjunction of features, such as a green square embedded within a display of red squares and green discs, response times increase with the number of distractors (Treisman & Gelade, 1980; Kristjánsson, Wang, & Nakayama, 2002; Wolfe, 1998).

Single-target visual search has been used to investigate the development of attention (e.g. Donnelly et al., 2007; Merrill & Conners, 2013; Merrill & Lookadoo, 2004; Taylor, Chevalier, & Lobaugh, 2003; Trick & Enns, 1998; Woods et al., 2013). Children up to about 6 years old have difficulty searching for targets defined by conjunctions of features (Donnelly et al., 2007; Trick & Enns, 1998; Woods et al., 2013). But when targets are defined by only a single feature, children’s search performance is quick and efficient regardless of distractor number, similar to adults (Donnelly et al., 2007; Merrill & Conners, 2013; Merrill & Lookadoo, 2004; Taylor et al., 2003; Trick & Enns, 1998; Woods et al., 2013). Conjunction search requires not only that observers find the two features defining the target, but they must also bind those features together (Treisman & Gelade, 1980; Treisman & Schmidt, 1982; see discussion in Johnson, Hollingworth, & Luck, 2008), which may place high demands on attention for children under the age of 6 years (Trick & Enns, 1998). Young children are also more susceptible to interference by distractors in conjunction search than adults, which has been attributed to the development of feature binding and voluntary attention shifts (Trick & Enns, 1998). Event-related potentials connected with attentional functioning are not fully mature until the age of 12 years (Taylor et al., 2003). Nevertheless, children’s conjunction search performance improves significantly when they are aged 6–7 years (Hommel, Li, & Li, 2004; Lobaugh, Cole, & Rovet, 1998; Merrill & Lookadoo, 2004), perhaps reflecting that top-down attentional control becomes more efficient.

### Visual foraging

Attention deployments in real-world situations are, however, rarely so simple that they involve a single target, where the task ends when the target is found. Foraging for multiple targets may involve a more dynamic way of measuring visual search and visual attention (Cain, Vul, Clark, & Mitroff, 2012; Hills et al., 2015; Jóhannesson, Thornton, Smith, Chetverikov, & Kristjánsson, 2016; Kalff, Hills, & Wiener, 2010; Klein & MacInnes, 1999; Kristjánsson, Jóhannesson, & Thornton, 2014; Smith, Hood, & Gilchrist, 2008; Wolfe, 2013). Such foraging studies have a long tradition in animal research and have focused on what kinds of behaviors lead to optimal foraging, or how animals can maximize their energy intake with as little effort as possible (see e.g. Benhamou, 2007; Hills, Kalff, & Wiener, 2013; for an overview, see Börger, Dalziel, & Fryxell, 2008; Pyke, Pulliam, & Charnov, 1977). These studies suggest that when food is easily found, predators randomly switch between different food types but when food is difficult to find, limited attentional capacities cause them to focus on a single food type and ignore other equally available items (Dukas, 2002). Recent evidence shows that human foraging behavior resembles that of animals in many ways. For example, they adapt their search strategy to the distribution of target items in the environment to optimize hit rate (see e.g. Cain et al., 2012; Kalff et al., 2010; Wolfe, 2013).

To study visual foraging as a function of age, we used a newly developed task by Kristjánsson et al. (2014). They studied visual foraging of adults using an iPad display consisting of 80 stimuli, from four stimulus categories, two of which were targets and two were distractors. The participants were instructed to find and tap all targets from both categories as quickly as possible, while avoiding tapping any distractors. While in most previous foraging studies visual salience was used to manipulate selection behavior (e.g. Bond, 1982; Dawkins, 1971), Kristjánsson et al. (2014) contrasted feature versus conjunction foraging to manipulate attentional load. In their feature condition, targets and distractors were defined by a single feature dimension (color), while in the conjunction condition, targets were defined by both color and shape. During feature foraging, participants randomly switched between target categories, while during conjunction foraging, most participants repeatedly selected targets from the same category (see also Jóhannesson et al., 2016). Kristjánsson et al. (2014) speculated whether this reflects that participants can simultaneously hold two features in working memory, while most participants cannot keep four features and their relation simultaneously in working memory. Interestingly, a small proportion of participants, termed “super-foragers,” could switch randomly between target categories during conjunction foraging, without either increases in error rates nor response times (see also Socé, Cain, & Wolfe, 2016, where observers switch rapidly between two target types during multiple-target search). These findings ran counter to predictions of theories which assume that only a single search template can be held in working memory at a time (see e.g. Huang & Pashler, 2007; Olivers, Peters, Houtkamp, & Roelfsema, 2011; van Moorselaar, Theeuwes, & Olivers, 2014). Working memory has, indeed, been shown to influence both visual search for pop-out targets and more difficult targets (Soto, Heinke, Humphreys, & Blanco, 2005; Soto, Humphreys, & Heinke, 2006). Kristjánsson, Saevarsson, and Driver (2013) showed how visual search performance is influenced by a concurrent visual working memory task among adults (see also Lee, Mozer, & Vecera, 2009) which has also been seen for verbal working memory (Soto & Humphreys, 2007).

### Executive functions (EF), self-regulation, and visual attention

Previous research suggests that executive functions (EF), which enable people to focus and maintain attention, remember instructions, and work on multiple tasks simultaneously, play a role in single target visual search (McClelland, Ponitz, Messersmith, & Tominey, 2010; Merrill & Conners, 2013; Woods et al., 2013; Zelazo & Müller, 2002), and that children’s difficulty with conjunction search may reflect underdeveloped executive functions (Woods et al., 2013). Mental planning and flexibility, working memory, and inhibition probably play a role in conjunction search by enabling participants to remember instructions, guiding spatial attention and keeping track of already searched locations. Woods et al. (2013) found that as organizational abilities in the visual search of children aged 2–18 years improved, so did conjunction search performance, probably reflecting the maturation of executive organizational processes. This is supported by the finding that 6-year-old children find it difficult to search for a singleton target in the presence of heterogeneous distractors (Merrill & Conners, 2013), suggesting that they lack the ability to maintain focus and ignore distractors. Feature search performance is not affected to the same degree as it may not rely on such executive processes (Merrill & Conners, 2013; Merrill & Lookadoo, 2004).

Foraging shares many characteristics with visual search and measuring executive functions is therefore likely to yield further insights into the development of foraging and visual attention abilities. Indeed, recent research suggests that there is a general neural search architecture in the brain that may represent a domain-general central executive search process, that may also play a role in foraging (Hills, Todd, & Goldstone, 2010).

A growing number of studies from multiple disciplines suggests that executive functions are primary cognitive constructs contributing to self-regulation (Barkley, 1997; McClelland et al., 2010). Self-regulation is a broad, multidimensional construct involving the control people have over their own cognition, emotions, and behaviors. As such, self-regulation involves the integration of executive functions enabling people to modify their behavior in order to navigate their social environment and reach their goals, such as in a challenging school environment (McClelland et al., 2010). Global measures of self-regulation are considered to reflect executive functions, including working memory, attention, and inhibition, so there should be considerable overlap in the characteristics of visual attention, executive functions, and self-regulation (Rueda, Posner, & Rothbart, 2005). The literature on the relation between these constructs is, nevertheless, surprisingly sparse (but see Büttner et al., 2014; Gerardi-Caulton, 2000; Sheese, Rothbart, Posner, White, & Fraundorf, 2008, van Hecke et al., 2012).

### The current study

Using the newly developed foraging task (Kristjánsson et al., 2014), we measured the foraging performance of children aged between 4:0 and 7:3 years, as previous studies suggest that between 6 and 7 years of age, children develop the ability to perform conjunction search (Hommel et al., 2004; Lobaugh et al., 1998; Merrill & Lookadoo, 2004). Furthermore, previous research suggests that executive functions and self-regulation develop rapidly at this age (see e.g. Blair, 2002; Vaszonyi & Huang, 2010).

The number of “runs” on each trial was our primary dependent variable. A run can be defined as “a succession of one or more types of symbols which are followed and preceded by a different symbol or no symbol at all” (Kristjánsson et al., 2014, p. 3). In our case, the symbols are the target items, which can be defined by a single feature (color) or a conjunction of two features (color and shape). Since there are two target categories and 40 targets per display, the minimum number of runs is 2 (if participants exhaustively cancel one category before switching to the other one) while the maximum is 40 (when a switch occurs after every single-target cancellation). During feature foraging, adults typically switch randomly between target categories, leaving the number of runs close to 20, but during conjunction foraging, most search each category exhaustively before turning to the next one, often completing trials in only two runs (Jóhannesson et al., 2016; Kristjánsson et al., 2014).

We had three main hypotheses. First, that as for adults, children’s run behavior would be close to random for feature foraging, while the number of runs would drop dramatically during conjunction foraging. Second, that the number of runs would increase with age during feature foraging. If adults can simultaneously hold two search templates in working memory, and run behavior can be used to estimate that ability, we expected 4-year-olds to have the largest difficulty with simultaneously holding two target categories in mind. Third, that self-regulation and working memory capacity (WMC) would be positively correlated with both number of runs and completed trials, but negatively correlated with response times for each target within a trial (intra-trial response times: ITRTs). If self-regulation involves the ability to purposely use several EFs simultaneously and working memory is an individual EF, then high global self-regulation scores should be correlated with the ability to use not only working memory but other helpful EFs such as inhibition (withholding from tapping distractors) and attentional flexibility leading to larger numbers of runs, lower ITRTs, and more completed trials.

## Method

### Participants

Forty-two kindergarteners and 24 first graders participated, aged 49–86 months (M = 68.15 months, SD = 11.69 months, 33 girls). For some analyses they were split into three age bins based on year of birth to compare groups. In Iceland, enrollment in kindergarten and school is based on the year of birth and children in the same age bin had all spent equal amounts of time in kindergarten/school, with the same curriculum. Every child in the oldest age group started school in the fall preceding testing (in March). All had normal or corrected to normal vision according to both their teachers and themselves. Approval from school administration was obtained, in addition to parental consent and verbal consent from each participant. All aspects of the experiment were reviewed and approved by the data protection authority and permission was granted by the Reykjavik Department of Education and Youth.

#### Foraging task

The foraging task from Kristjánsson et al. (2014) was administered. The Spearman-Brown coefficient for the children’s data (using inter-target response times) was 0.843 indicating good reliability for the task.

##### Equipment

The stimuli were displayed on an iPad 2 with screen dimensions of 20 × 15 cm and an effective resolution of 1024 × 768 pixels, placed on a table in front of the participant in landscape mode, so that viewing distance was approximately 50 cm (this varied slightly as the younger children, especially, tended to move around while performing the task). Stimulus presentation and response collection were carried out by a custom iPad application written in objective-C using Xcode and Cocos2d libraries.

##### Stimuli

**Fig. 1:**
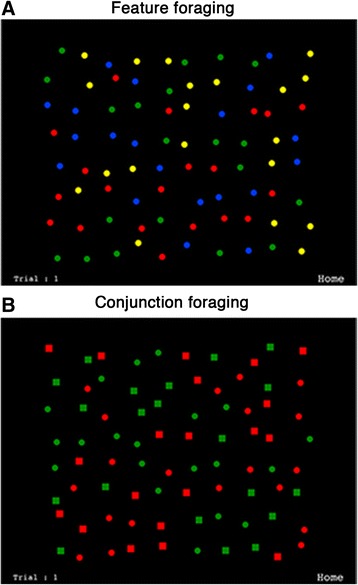
Examples of the iPad foraging trials. Panel (**a**) shows the feature condition, where the task is to tap all *red* and *green circles* while ignoring *blue* and *yellow* (or vice versa). Panel (**b**) shows the conjunction condition where the task is to tap all the *red squares* and the *green circles* (or vice versa)

Each trial consisted of 80 colored items randomly distributed on the screen. For feature-foraging, the targets were red and green disks and the distractors were yellow and blue disks for half of the children, while for the other half this was reversed. For conjunction foraging, the targets were red squares and green disks and the distractors were either green squares and red disks for half of the trials or the reverse. Each trial had 40 target items and 40 distractors. There were 20 stimuli in each group, drawn on a black background (see Fig. 1). The diameter of targets and distractors was 20 pixels (approximately 0.46° at 40 cm distance). The items were randomly distributed on a non-visible 10 × 8 grid that was offset from the edge of the screen by 150 × 100 pixels. The viewing area therefore occupied 15 × 12 cm (approximately 17.1 × 13.7°). The position of individual items within the grid was jittered by adding a random horizontal and vertical offset while gaps between rows and columns ensured that items never approached or occluded each other. The overall spatial layout and the location of targets and distractors was randomly generated for each trial.

##### Data analyses

As in Kristjánsson et al. (2014; see also Jóhannesson et al., 2016), both ITRTs and the number of runs on each trial were measured. ITRT denotes the time between each tap on the target items. A run is defined as consecutive tapping of the same target type, one or more times; preceded and followed by tapping a different target type or no tap (Kristjánsson et al., 2014). The number of runs ranges from 2 (every single target of a specific type is cancelled before observers start with the other category) to 40 (observers always switch between item categories). The number of runs is therefore the inverse of how often a participant switches between target types within a trial. When switches between target categories are completely random, the average number of runs should be just above 20.

#### Behavioral self-regulation task

The Head-Toes-Knees-Shoulders task (HTKS) was used to measure self-regulation abilities. The HTKS measures broad aspects of self-regulation, including working memory, attention, and inhibition (Ponitz et al., 2008; Ponitz, McClelland, Matthews, & Morrison, 2009), showing high reliability and validity in recent studies (e.g. Gestsdóttir et al., 2014; Ponitz et al., 2008, 2009). The task consists of 30 trials, preceded by a few rehearsal trials. The first ten trials involve two commands (“touch your head” and “touch your toes”). The children are instructed to do the opposite of what the examiner tells them to do, so if the examiner tells them to touch their head they should touch their toes. In the second part, two new commands are added (“touch your shoulders” and “touch your knees”). In the third part, the rules are reversed so now head is paired with knees and shoulders are paired with toes. The children received zero points for an incorrect response, one point for a self-corrected response, and two points for a correct response. Scores on the HTKS range from 0 to 60, with higher scores indicating better self-regulation. In each part of the test, administration is discontinued if the child is not able to perform at least five actions correctly (either correct or self-corrected). This Icelandic version has been translated and back-translated from the original version, and has been used in prior studies of self-regulation in Iceland (e.g. Birgisdóttir, Gestsdóttir, & Thorsdóttir, 2015; Gestsdóttir et al., 2014).

#### Working memory task

The *Sentences* subtest from the WPPSI-R^IS^ (Guðmundsson & Olafsdóttir, 2003) was used to assess WMC. The subtest measures the storage ability of verbal working memory and has high reliability and validity (Guðmundsson & Olafsdóttir, 2003). The children had to repeat increasingly long and complicated sentences read by the examiner. Errors such as skipping a word, adding words, adding or skipping endings of words and mixing the order of the words, were counted. The sentences subtest of WPPSI-R^IS^ measures the storage ability of the working memory, primarily such constructs as the episodic buffer and the phonological loop (Baddeley, 2000). A verbal working memory test was used to attempt to dissociate the constructs being measured as visual working memory measures might rely more heavily on visual attention which might contribute directly to foraging.

### Procedure

Each session lasted about 20 min, and took place in a quiet room with normal illumination. The participants finished the foraging, sentences, and HTKS tasks in counterbalanced order. During foraging, participants were instructed to tap all targets as quickly as possible using their right index finger. A picture showing the targets was placed next to the iPad and the colors and the shapes were read out loud to the children (“You have to tap all the red circles and all the green circles. You can tap them in any order you like, and if you forget which colors you are supposed to tap, look at this picture to remind yourself”). The targets disappeared immediately following the tap. If participants tapped a distractor, the trial ended, an error message was given, and a new trial started. Each participant performed nine trials, one training trial followed by four trials of each of the two tasks in counterbalanced order. In total, each participant attempted to complete four feature foraging trials and four conjunction foraging trials. One trial involves a completed sequence where all 40 targets were tapped. Pilot tests showed that if the children could not complete a single trial after three attempts, the task was too complicated for them. If participants did not complete a single trial in five attempts, testing was discontinued.

## Results

### Foraging patterns

**Fig. 2:**
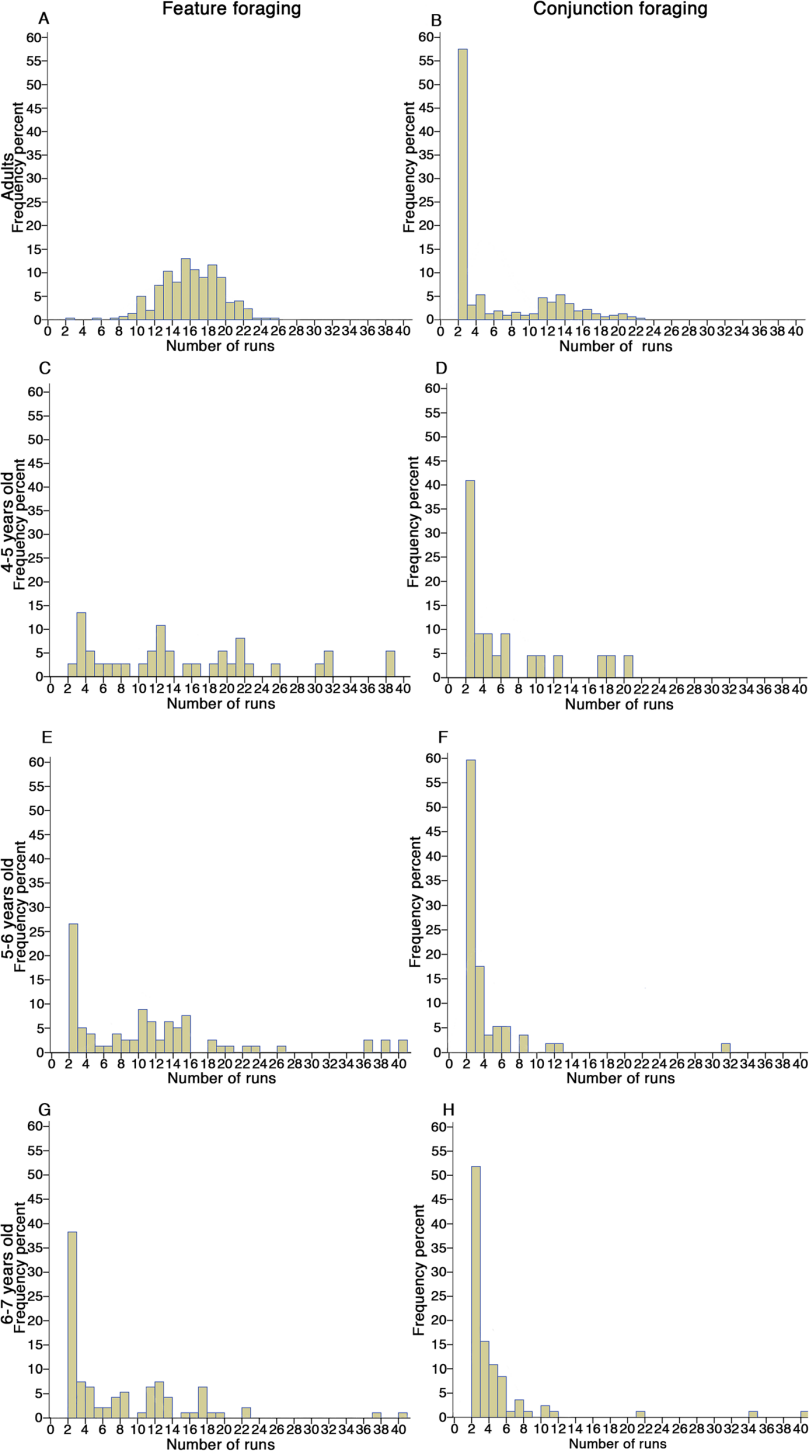
The number of runs during feature (**a**, **c**, **e**, **g**) and conjunction (**b**, **d**, **f**, **h**) foraging for the different age groups. Adult performance from Kristjánsson et al. (2014) is shown for comparison in the *top row*, 4-year-olds in the *second row*, 5-year-olds in the *third row*, and 6-year-olds in the *bottom row*

Foraging patterns for children (shown in Fig. 2) differ notably from the adult results (replotted from Kristjánsson et al. (2014) in Fig. 2a and b). The differences between feature and conjunction foraging are much less pronounced for children than adults. Figure 2c and d show the number of runs for the youngest age group, 4–5 years old, for feature and conjunction foraging, respectively. Conjunction foraging is similar to the adult pattern (Fig. 2b and d) with a peak at two runs. The feature foraging pattern differs strongly from the adult pattern, however (Fig. 2a and c). The highest peak in Fig. 2c is at three runs and roughly one-third of trials is completed in less than ten runs, while adults rarely used less than ten runs (Fig. 2a).

The pattern in Fig. 2e and f for the 5–6-year-olds and in Fig. 2g and h for the 6–7-year-olds is similar. There are high peaks at two runs for both feature and conjunction foraging, yet when looking at the percentages the peak is more pronounced for the conjunction condition. During feature foraging, a far higher portion of trials consists of two runs and a much higher portion of trials involve less than ten runs for the children than for adults.

Note that even though the patterns look similar between age groups, the percentages reveal interesting differences. During feature foraging (where adults rarely use two or three runs), 4–5-year-olds use two to three runs on 16.2% of trials, 5–6-year-olds on 31.6% of the trials, and 6–7-year-olds on 45.7% of the trials, moving gradually away from the adult pattern.

An ANOVA confirmed this difference in number of runs during feature foraging between the three age groups and adults using average number of runs for each participant *F*(3,77) = 6.037 *p* = 0.001 but a non-significant difference for conjunction foraging, *F*(3,69) = 0.347 *p* = 0.791. A Bonferroni post-hoc analysis revealed that the significant difference is between adults and 6-year-olds *p =* 0.005 and between 4- and 6-year-olds *p* = 0.005. Since run numbers are far from normally distributed, especially for conjunction foraging, a non-parametric test was conducted. A Kruskall–Wallis test confirmed the results from the ANOVA, a significant difference between the age groups for feature (χ2(3) = 22.15 *p* < 0.001) but not conjunction foraging (χ2(3) = 1.93 *p* = 0.587). Critically, this difference between adult and children’s performance is not captured by single-target visual search tasks.

HTKS and WMC scores were binned so they each formed four groups with equal participant numbers. ANOVAs did not reveal any significant effects of self-regulation or WMC upon run number for either feature or conjunction foraging.

### Intra-trial response times

Average ITRTs for each participant were calculated as the time between taps of each target stimulus by subtracting the time of the preceding tap from the current tap time. A multivariate ANOVA confirmed that age had a significant effect on average ITRTs for both feature and conjunction foraging (*F*(2,53) = 13.18 *p* < 0.001, *F*(2,53) = 10.92 *p* < 0.001, respectively) with older participants being faster than the younger ones (6 < 5 < 4). There was also a significant effect of HTKS scores on average ITRTs during feature foraging and a marginally significant effect during conjunction foraging (*F*(3,53) = 3.94 *p* = 0.013 and *F*(3,53) = 2.46 *p* = 0.074, respectively), Participants with higher HTKS scores were faster than participants with lower HTKS scores. There was also a significant effect of WMC scores on average ITRT for feature (*F*(3,53) = 3.36 *p* = 0.026) but not conjunction foraging (*p* = 0.159), where participants with higher WMC scores were faster than participants with lower WMC scores. This indicates that higher WMC and self-regulation scores are more strongly correlated with ITRTs during feature than conjunction foraging.

**Fig. 3:**
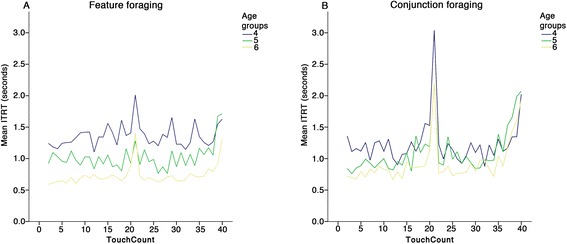
Average RTs for each target tapped throughout the trials during feature (**a**) and conjunction (**b**) foraging. Separate lines show the different age groups

For ITRTs throughout trials (Fig. 3), feature foraging ITRTs decrease with age. The ITRTs are relatively flat throughout the trial with two exceptions. First, there is a rise in ITRTs at the 21st tap, which is expected if participant use two runs, as this is where they would switch between categories. This rise is especially apparent for 6-year-olds, consistent with previous results. Second, there is a rise in ITRTs towards the end of trials, especially for the 5- and 6-year-olds. This rise probably reflects problems with finding the last few targets. When there is only one target left, there are 40 distractors and one target on the screen. The pattern is similar for conjunction foraging. The difference throughout the trial between the age groups is not as clear, but the rise in ITRTs at the middle of the trial and towards the end of it is more pronounced.

**Fig. 4:**
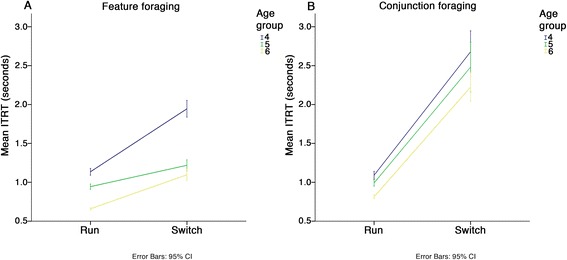
Average RTs depending on whether a tap was a switch between target categories (Switch = 1) or a repeat from the previous tap (Switch = 0) in feature (**a**) and conjunction (**b**) foraging. *Separate lines* show the different age groups. *Error bars* denote the 95% confidence intervals

Higher ITRTs at the middle of the trials (Fig. 3) suggest that switching between target categories is markedly harder than repeating previous selections (see e.g. Brascamp, Blake, & Kristjánsson, 2011; Chetverikov & Kristjánsson, 2015). Figure 4 shows clear switch-costs in ITRTs when tapping a stimulus from a different target category than the previous tap (switch) compared to ITRTs when tapping a stimulus from the same target category as the last tap (run). This cost is much larger for conjunction foraging. A mixed-design repeated measures ANOVA, using average ITRTs for each participant confirms this. The main effects of condition and switch were both significant (*F*(1,45) = 109.26 *p* < 0.001 and *F*(1,45) = 122.22 *p* < 0.001) as was the interaction, indicating greater switch costs during conjunction foraging (*F*(2,45) = 71.73 *p* < 0.001). The between-subject factor of age was significant (*F*(2,45) = 7.099 *p* = 0.002) and a Bonferroni post-hoc test confirmed significant differences between the 4- and 5-year-olds and 4- and 6-year-olds (*p* = 0.025 and *p* = 0.002). There was no interaction between age group and switch-costs or condition.

**Fig. 5:**
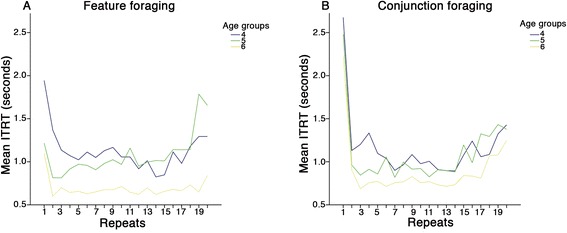
Average RTs for different number of repeats, one repeat being a switch, for feature (**a**) and conjunction (**b**) foraging. *Separate lines* show the different age groups

Figure 5 shows how ITRTs change the more often the same target is tapped. The first tap from any target category (switch) is slow as shown. Otherwise, the ITRTs are relatively flat throughout the run with a rise towards the end that is larger for conjunction than feature foraging. This is especially true for the 6-years-olds whose ITRTs are flat from repeat number 2 until the slight increase for the last repeat during feature foraging. During conjunction foraging the ITRTs increase earlier or around repeat number 16 or 17. This could reflect difficulty differences between the two tasks. A mixed-design repeated measures ANOVA was conducted with average ITRT for each participant at each repeat in each condition (with the exception of repeat 1, which is a switch and has been shown to differ from repeats above). The main effect of repeats was not significant (not surprisingly as it is a linear model), but the within-subject contrast for repeat was significant with a quadratic model (*F*(1,45) = 6.65 *p* = 0.013). The interaction between age and repeats was marginally significant (*F*(38,810) = 1.35 *p* = 0.086). The main effect of condition was significant (*F*(1,45) = 19.11 *p* < 0.001) and the interaction between age and condition was marginally significant (*F*(2,45) = 3.47 *p* = 0.075). Both the interactions between repeats and conditions and the three-way interaction between age, repeats, and condition were significant (*F*(18,810) = 2.75 *p <* 0.001 and *F*(36,810) = 1.46 *p =* 0.042, respectively) which confirms that the rises in ITRTs do not reflect random performance fluctuations. The between-subject variable of age was significant (*F*(2,45) = 8.23 *p* = 0.001) and a Bonferroni post-hoc test confirmed that the 6-years-olds differed significantly from the 4- and 5-year-olds (*p =* 0.002) while there was no difference between 4- and 5-year-olds (*p* = 0.025). A potential problem with interpreting the rises towards the end of long runs is that the number of taps behind the higher repeats is lower than the lower number of repeats as only trials with 2 runs will have a tap at 20 repeats. This could mean that participants that were more likely to complete a trial with 2 runs were the participants that were slower and that faster participants do not contribute to the average at the higher repeats. This explanation is, however, unlikely. The clear switch-cost shown across all age groups, especially for conjunction foraging, should then produce faster average ITRTs for participants that do not switch often, leading to faster average ITRTs on trials with more repeats. The fact, that the ITRTs are slower, not faster, for the highest number of repeats indicates that this simply reflects difficulty in finding the last targets in a category.

### Completed trials

Multivariate ANOVAs were used to investigate effects of each independent variable upon the total number of completed trials. First, age had a significant effect on the number of completed trials, for both feature and conjunction foraging (*F*(2,66) = 14.80 *p* < 0.001 and *F*(2,66) = 13.23 *p* < 0.001, respectively). A Bonferroni post-hoc analysis showed a significant difference between 6-year-olds versus 4- and 5-year-olds (*p* < 0.001 and *p* = 0.011, respectively) with a non-significant difference between the 4- and 5-year-olds (*p* = 0.254).

Self-regulation scores also affected the number of completed trials, both during feature and conjunction foraging (*F*(3,66) = 7.90 *p* = 0.002 and *F*(3,66) = 15.42 *p* < 0.001, respectively). Bonferroni post-hoc analyses showed that the lowest bin, the 25% of participants with the lowest self-regulation scores, completed significantly fewer trials than the top two bins (*p* = 0.007 between bin 1 and 3, *p* = 0.004 between bin 1 and 4) during feature foraging, with no other significant differences. The pattern was identical during conjunction foraging (*p* < 0.001 between bin 1 and 3, *p* = 0.004 between bin 1 and 4).

WMC scores significantly affected the number of completed trials during feature (*F*(3,65) = 2.89 *p* = 0.043) but not conjunction foraging (*p* = 0.143). A Bonferroni post-hoc analysis did not reveal the significant effect, but the lowest bin probably separates itself from the others (*p* = 0.084 between bins 1 and 2, *p* = 0.122 between bins 1 and 3 and *p* = 0.153 between bins 1 and 4).

### Correlations

Finally, we measured whether WMC scores and HTKS scores were positively correlated with both run number and completed trials, but negatively correlated with ITRTs using a partial correlation table (Table 1). Global self-regulation and WMC scores and the following dependent variables: number of runs (NR), response times (RT), and completed trials (CT) for both foraging conditions, were inserted. Age was a control variable so that the correlation between the dependent and independent variables could be assessed without the expected correlation due to age-related abilities.

**Table 1:** Partial correlation table corrected for age

Control variable		WMC	Mean NR- feature	Mean NR- conjunct	Mean RT- feature	Mean RT- conjunct	CT- feature	CT- conjunct
Age	HTKS	0.311*	0.097	0.061	–0.454**	–0.271*	0.075	0.149
	WMC		0.048	0.096	–0.298*	–0.160	0.145	0.138
	Mean NR- feature			0.685**	0.188	0.225*	–0.107	–0.461**
	Mean NR- conjunction				0.189	0.370*	0.003	–0.319*
	Mean RT- feature					0.700**	–0.113	–0.221
	Mean RT- conjunction						–0.174	–0.194
	CT- feature							0.531**

*p < 0.05; **p < 0.001

*HTKS* head, toes, knees and shoulders which is the global self-regulation measure, *WMC* working memory capacity, *NR* number of runs, *RT* reaction time, *CT* completed trials

Self-regulation scores were significantly correlated only with WMC scores and ITRTs for both feature and conjunction foraging. WMC scores were only significantly correlated with ITRTs during feature foraging. This contrasts with previous analyses where HTKS scores and WMC scores were correlated with completed trials. This suggests that age, which was controlled for in the partial correlation table, might explain effects in earlier ANOVAs. A multivariate ANOVA with completed trials during feature and conjunction foraging as the dependent variables, age (binned by year of birth) as the fixed factor, and HTKS and WMC scores as covariates tested this. The results suggest that age does indeed have a strong effect on the number of completed trials (*F*(2,65) = 7.35 *p* < 0.001 for feature and *F*(2,65) = 8.04 *p* < 0.001 for conjunction foraging). The HTKS effect was still significant both for the completed trial number during feature and conjunction foraging (*F*(1,65) = 5.05 *p* = 0.028, feature and *F*(1,65) = 8.25 *p* = 0.006 conjunction). The effect for the WMC scores is weaker, with the effect for feature foraging only marginally significant and the effect for conjunction foraging still not significant (*F*(1,65) = 3.12 *p* = 0.082, feature and *F*(1,65) = 0.95 *p* = 0.334, conjunction). The effect for HTKS scores on the number of completed trials is still there, even when controlling for age, albeit weaker.

Perhaps the most interesting results in Table 1 are the correlations between the dependent variables. ITRTs and completed trials during conjunction foraging were correlated with run number during both feature and conjunction foraging. Neither ITRTs nor completed trials during feature foraging correlated with run number. The slower the ITRTs during conjunction foraging, the more often participants switched between target categories during both feature and conjunction foraging. Furthermore, the more trials the children managed to complete during conjunction foraging, the fewer switches they made between target categories, both during feature and conjunction foraging. This fits well with the switch-cost analysis which showed an interaction between switch-costs and condition. If switch-costs are higher during conjunction foraging, then the average ITRTs would be expected to be higher for those participants that switch more often.

## Discussion

Children’s foraging patterns differed markedly from those previously observed among adults (Jóhannesson et al., 2016; Kristjánsson et al., 2014). The children had trouble foraging for targets defined by two different features which adults have little trouble with. Importantly, such feature versus conjunction differences for children are not seen in single-target search tasks, which are most often used to assess the development of visual attention, showing the value of this new foraging task for testing cognitive development in children.

Second, as children got older, more trials consisted of only two runs, a pattern that has previously been interpreted as a difficulty with holding two target categories simultaneously in mind (Jóhannesson et al., 2016; Kristjánsson et al., 2014). We speculate that underdeveloped metacognition, an important aspect of self-regulation (Clerc, Miller, & Cosnefroy, 2014), may explain this opposite pattern to what we expected. Although recent evidence suggests that children aged as young as 5 years (Vo, Li, Kornell, Pouget, & Cantlon, 2014), and even 3 years (Destan, Hembacher, Ghetti, & Roebers, 2014), have developed rudimentary metacognition, they tend not to be very good at using their prior performance to adapt to tasks through repeated exposure and tend to be overconfident about their performance (Finn & Metcalfe, 2014). During foraging, the oldest children may have cancelled every target from one category before turning to the next, failing to realize that this might not be the most effective way of cancelling all targets. A number of participants indeed voiced their intent of completing one target category before the other during feature foraging. Almost 40% of the oldest children’s feature foraging trials consisted of only two runs, a considerably higher percentage than for the younger children.

### Working memory

Kristjánsson et al. (2014) discuss the possibility that adult participants can simultaneously hold two feature values in working memory and can therefore perform feature foraging by picking targets from the two categories at random, perhaps selecting the target closest to the last one. When load increases as selection becomes based on conjunctions of features, a more efficient strategy may be to pick all the targets from one target category before turning to the other one. Based on those speculations (see also Kane, Poole, Tuholski, & Engle, 2006; Soto & Humphreys, 2007) we predicted that better working memory would lead to more runs for feature foraging. The fact that working memory seemed not to affect run number may be because the *Sentences* subtest of WPPSI-R^IS^ is a measure of verbal, not visual, working memory. We used a verbal task instead of a visual one to measure WMC independently of visual attentional abilities. Using a different measure of working memory in future studies should further clarify the role of working memory in foraging. Importantly, however, there was a relationship between working memory and the number of completed trials (see below).

### Intra-trial response times and completed trials

We expected ITRTs to decrease with age and self-regulation scores to correlate with ITRTs, as greater self-regulation abilities may indicate less distractibility and greater organizational skills, leading to faster responses. As expected, the oldest children had the lowest ITRTs. In addition, both global self-regulation and working memory significantly affected ITRTs, especially during feature foraging.

We expected the strongest effects of global self-regulation and working memory upon the number of completed trials, since this is the only dependent variable that incorporates performance of those who did not complete a single feature or conjunction trial. Leaving those participants out might lead to skewed results, if the hypothesis that self-regulation contributes to foraging performance is correct. Self-regulation did indeed correlate with the number of completed trials, for both feature and conjunction foraging, and further analyses showed that participants with the lowest self-regulation scores completed the fewest trials. This suggests that basic self-regulation abilities are necessary to complete the foraging tasks. Furthermore, working memory affected the number of completed trials for feature, but not conjunction foraging, where the children with the lowest working memory ability completed fewer trials than their peers. These results indicate that global self-regulation ability and individual EFs relate to children’s foraging performance.

### Intra-trial response times

An advantage of our foraging task is that we can assess how performance progresses throughout trials. Switching between target categories within trials slows performance. There was an indication that ITRTs became slower towards the end of trials. The fact that this slowing was much greater for conjunction foraging suggests that slowed ITRTs reflect difficulties in finding the last few stimuli among 40 distractors, in line with results from single target studies showing that set-size effects are larger for conjunction than feature search (e.g. Nordfang & Wolfe, 2014; Treisman & Gelade, 1980). Switch costs between target categories were larger for conjunction than feature foraging, but there was no interaction between age and switch costs, so they are relatively stable during those years and mirror the results from adults (Jóhannesson et al., 2016).

## Conclusions and future directions

We investigated foraging, self-regulation, and verbal working memory. Foraging performance was related both to self-regulation and working memory and we suggest that the foraging task may be developed further to work as a proxy for assessing cognitive development. The role individual EFs, or subcomponents of self-regulation, play in foraging should be measured for this purpose. For example, it seems likely that inhibition helps in avoiding distractors and run number seems to increase with more attentional flexibility. Increased attentional flexibility could also lead to a change in foraging strategies if the current one is not efficient. Switching strategies also requires that participants realize that the current strategy is not optimal. This requires metacognition, which would therefore be a useful ability to assess.

Children have trouble foraging for targets defined by two different features. Adults have little trouble with this, suggesting that children have not developed the necessary skills to select and implement the most appropriate strategies. The fact that the foraging pattern moves away from the adult pattern with age may reflect increased ability to form strategies, combined with an inability to implement them and underdeveloped insight into their own performance. To assess how foraging patterns develop into adulthood, foraging should be tested for a larger age group to assess how and when foraging patterns start to resemble those of adults, providing important information about the development of visual attention. Our visual foraging task is easy to administer and interpret and has potential for shedding light on the development of visual attention and cognitive development more generally.
